# Comparison of Automated Thresholding Algorithms in Optical Coherence Tomography Angiography Image Analysis

**DOI:** 10.3390/jcm12051973

**Published:** 2023-03-02

**Authors:** David Prangel, Michelle Prasuhn, Felix Rommel, Salvatore Grisanti, Mahdy Ranjbar

**Affiliations:** 1 Laboratory for Angiogenesis & Ocular Cell Transplantation, Ratzeburger Allee 160, 23538 Lübeck, Germany; 2 Department of Ophthalmology, University Hospital Schleswig-Holstein, University of Luebeck, Ratzeburger Allee 160, 23538 Luebeck, Germany

**Keywords:** optical coherence tomography angiography (OCTA), automated thresholding, binarization, image processing

## Abstract

(1) Background: Calculation of vessel density in optical coherence tomography angiography (OCTA) images with thresholding algorithms varies in clinical routine. The ability to discriminate healthy from diseased eyes based on perfusion of the posterior pole is critical and may depend on the algorithm applied. This study assessed comparability, reliability, and ability in the discrimination of commonly used automated thresholding algorithms. (2) Methods: Vessel density in full retina and choriocapillaris slabs were calculated with five previously published automated thresholding algorithms (Default, Huang, ISODATA, Mean, and Otsu) for healthy and diseased eyes. The algorithms were investigated with LD-F2-analysis for intra-algorithm reliability, agreement, and the ability to discriminate between physiological and pathological conditions. (3) Results: LD-F2-analyses revealed significant differences in estimated vessel densities for the algorithms (*p* < 0.001). For full retina and choriocapillaris slabs, intra-algorithm values range from excellent to poor, depending on the applied algorithm; the inter-algorithm agreement was low. Discrimination was good for the full retina slabs, but poor when applied to the choriocapillaris slabs. The Mean algorithm demonstrated an overall good performance. (4) Conclusions: Automated threshold algorithms are not interchangeable. The ability for discrimination depends on the analyzed layer. Concerning the full retina slab, all of the five evaluated automated algorithms had an overall good ability for discrimination. When analyzing the choriocapillaris, it might be useful to consider another algorithm.

## 1. Introduction

Optical coherence tomography angiography (OCTA) is a non-invasive imaging modality that provides high-resolution, depth-resolved images of the chorioretinal blood flow [1,2]. OCTA-based vessel density (VD) has been proposed as a promising imaging parameter and biomarker in various clinical studies, in which it has been used to discriminate healthy eyes from diseased ones such as in age-related macular degeneration (AMD), diabetic retinopathy (DR), uveitis, and retinal vein occlusion (RVO) [3,4,5,6].

The calculation of VD is quite heterogeneous: manual, semiautomated, automated thresholding algorithms, fixed thresholds, and machine learning approaches can be applied [5,7,8,9,10]. A study by Rabiolo et al. found significant differences in the determined VD between automated and manual methods [11]. Advantages of automated methods over manual algorithms in terms of repeatability and detection of macular pathologies were found in a recent study by Terheyden et al. [12]. Therefore, image processing with automated thresholding appears more promising. Yet, further evaluation is necessary. The different thresholding algorithms can generally be divided into three main groups: firstly cluster-based algorithms such as Otsu, which uses an analysis of variance to split the image into two separate parts, and Default and ISODATA, where clustering is a dynamic process consisting of five sub-steps based on the K-means algorithm; secondly the Mean algorithm, which is a simple histogram-based algorithm, using the mean grey value as the threshold for image binarization; thirdly, Huang uses Shannon’s entropy for image binarization and is therefore entropy-based.

It is of high importance to understand differences and errors in the applied methods. Because OCTA has become an important modality research, but also a clinical routine, it is relevant to achieve comparable results and to apply the methods in a correct and standardized manner. Automated thresholding aids analysis of possible parameters such as VD and therefore needs to be well understood for the various disease entities and devices. Herein, we assess the comparability of five commonly used automated thresholding algorithms regarding reliability, agreement, and ability to discriminate healthy eyes from diseased ones focusing on the retina as well as the choriocapillaris.

## 2. Materials and Methods

Electronic clinical records (Orbis, Agfa Health-Care GmbH; Bonn, Germany) and SD-OCTA (Copernicus Revo NX130; Optopol Technology Ltd., Zawiercie, Poland) images from patients with retinal vein occlusion (RVO), diabetic retinopathy (DR), Uveitis, and neovascular age-related macular degeneration (AMD), who were already enrolled in various other studies and attended our facility from 24 April to 10 May 2019, were reviewed. These studies were approved by the ethics committee of the University of Lübeck, Germany (vote reference #18-102, 18-103 and 19-335). At the time of image acquisition, there was no intra- or subretinal fluid present. No affected eyes of patients with uveitis and RVO were assigned to the control group. General inclusion criteria were age ≥18 years, spherical and cylindrical aberration of ±3 and ±1 diopters, respectively, and 5 × 5 mm OCTA scans with a signal strength ≥ 8. Exclusion criteria were motion and other artifacts on OCTA images as well as the presence of pathological ocular conditions other than RVO, DR, Uveitis, and AMD [13]. Angiograms were taken at the same time of day to avoid distortion due to diurnal changes [14].

Three OCTA images per eye were consecutively obtained using the SD-OCTA device, which operates at 130,000 A-scans per second and a central wavelength of 840 nm. The axial resolution of the system is 5 µm and the transverse resolution 12 µm in tissue. The choriocapillaris angiograms were generated by manually measuring a 20 µm slab starting from the automated RPE segmentation. En face images (512 × 512 pixel) of the full retina slab (superficial and deep retinal layer) and choriocapillaris slab were exported in PNG (Portable Network Graphics) format.

**Figure 1:**
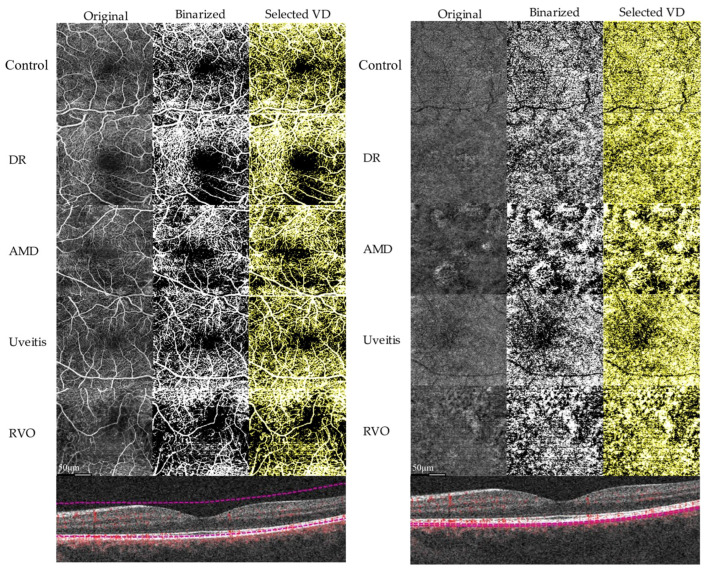
Image processing and vessel density (VD) calculation using the Mean algorithm as an example for the groups control, diabetic retinopathy (DR), age-related macular degeneration (AMD), Uveitis, and retinal vein occlusion (RVO) eyes in full retina angiograms (**left**), and choriocapillaris angiograms (**right**). The respective B-scans below show the segmentation for these layers.

ImageJ (NIH, Version 1.52q, Bethesda, Rockville, MD, USA), an open-source image processing software, was used for image analysis. The OCTA images were converted to 8-Bit format and binarized with five automated thresholding algorithms (Default, Huang, ISODATA, Mean, and Otsu) implemented in ImageJ. Vessel density was calculated based on the results of image binarization for white pixels in relation to all pixels of an image as previously reported (Figure 1) [15].

All statistical analyses were performed with SPSS Statistics, version 24 (IBM Corporation, Armonk, NY, USA) and R software (version 3.6.3, R Foundation for Statistical Computing, Vienna, Austria). A *p*-value of <0.05 was considered statistically significant. Data were tested for normality with the Shapiro–Wilk test. As OCTA data were found not to be distributed normally, differences between the five automated thresholding algorithms were evaluated with non-parametric testing using LD-F2 analysis [16]. An LD-F2 analysis uses robust rank-based statistics for longitudinal data and small sample sizes in factorial experiments. This study has a two-factorial design in which the eyes of the same patient as one factor and the use of the different algorithms on the same population as the second were included in statistical analysis. Intra-algorithm reliability between the three OCTA images of each eye was evaluated with intraclass correlation coefficients (ICCs). ICC values less than 0.5 indicate poor reliability, values between 0.5 and 0.75 indicate moderate reliability, values between 0.75 and 0.9 indicate good reliability, and values greater than 0.90 indicate excellent reliability [17]. For inter-algorithm agreement, Bland–Altman plots with the limits of agreement (LoA) set at 1.96 standard deviations (SDs), which results in a 95 % confidence interval (CI), were evaluated [18]. The ability to discriminate healthy eyes from disease-affected eyes (DR, RVO, Uveitis, and AMD) in full retina and choriocapillaris slabs was evaluated with receiver operating characteristic (ROC) curves and area under the curve (AUC) values [19,20].

## 3. Results

A total of 91 eyes of 51 patients were enrolled in this study. Demographic and clinical data are reported in Table 1. Twenty-four (47.3%) male and twenty-seven (52.7%) female participants were included in this study, with a mean age of 70.5 years.

**Figure 2:**
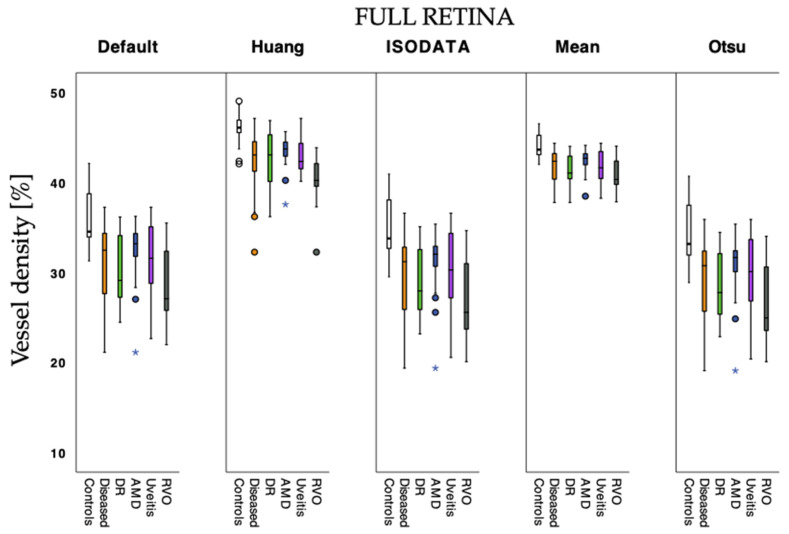
Vessel density values calculated with the tested algorithms for control and diseased eyes in full retina angiograms. Subgroups of diabetic retinopathy (DR), age-related macular degeneration (AMD), uveitis, and retinal vein occlusion (RVO) were also considered. Circles: outliers of 1.5 times the interquartile range of quartile 1 or quartile 3; stars: extreme outliers of 2.5 times the interquartile range of quartile 1 or quartile 3, respectively.

**Figure 3:**
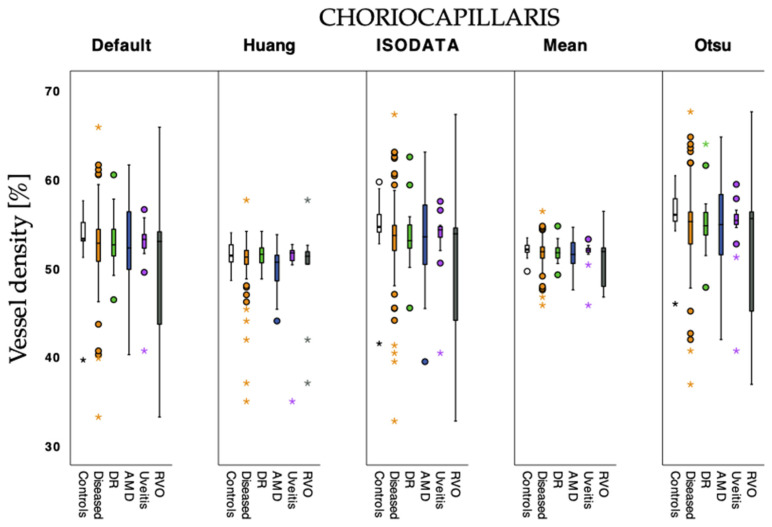
Vessel density values calculated with the tested algorithms for control and diseased eyes in choriocapillaris angiograms. Subgroups of diabetic retinopathy (DR), age-related macular degeneration (AMD), uveitis, and retinal vein occlusion (RVO) were also considered. Circles: outliers of 1.5 times the interquartile range of quartile 1 or quartile 3; stars: extreme outliers of 2.5 times the interquartile range of quartile 1 or quartile 3, respectively.

Figure 2 and Figure 3 show VD values estimated with the different algorithms as a comparison between groups. Default, ISODATA, and Otsu estimated lower VD values than Huang and Mean in the full retina slab (Figure 2). In the choriocapillaris slabs, estimated VD values differed only slightly, regardless of which algorithm was used (Figure 3). Vessel density differed significantly between the different algorithms for the full retina and choriocapillaris slabs in the LD-F2-analysis (*p* < 0.001).

In Table 2 and Table 3, intra-algorithm values of full retina and choriocapillaris angiograms are reported.

Concerning full retina values, Default, Otsu, and ISODATA had excellent reliability for healthy control eyes (ICC > 0.9), while a good reliability (ICC > 0.75) was observed for Mean and Huang. Diseased eyes in total had a good reliability (ICC > 0.75), except for Huang, which only had a poor reliability (ICC < 0.5). An examination of the various subgroups of diseased eyes indicates that all algorithms had an excellent reliability for eyes with DR. In AMD eyes, reliability was only moderate using all five algorithms. Excellent reliability was detected in eyes with uveitis and RVO, except for the Huang algorithm, which had only moderate reliability in uveitis and no reliability in RVO eyes (Table 2).

In choriocapillaris slabs, healthy control eyes had only a poor reliability with all algorithms (ICC < 0.5) except for Huang, which showed a moderate reliability (ICC > 0.5). Diseased eyes had an excellent reliability with Default, ISODATA, and Otsu (ICC > 0.9). Huang und Mean showed a good reliability (ICC > 0.75). Looking into the various subgroups of diseased eyes, Huang had a good reliability in DR and AMD (ICC > 0.75), while all other algorithms had an excellent reliability (ICC > 0.9). All algorithms delivered an excellent reliability in uveitis and RVO eyes (Table 3).

Table 4 and Table 5 show the results of the Bland–Altman analysis for the inter-algorithm agreement of the full retina and choriocapillaris angiograms. In the full retina slabs, mean difference (MD) and limits of agreement (LoA) were wider, which indicates a lower level of agreement between algorithms. Default, Otsu, and ISODATA had a good agreement. All other algorithms had a poor agreement, both in the full retina and choriocapillaris slabs.

**Figure 4:**
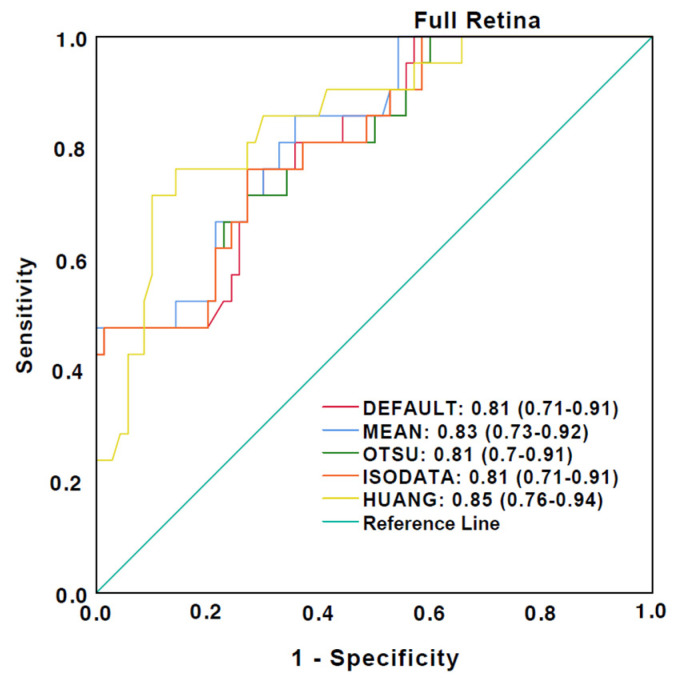
Receiver operating characteristics (ROC) curves for discrimination of diseased eyes from the healthy control group in the full retina slabs. The caption shows the area under the curve (AUC) values and the 95% confidence interval.

**Figure 5:**
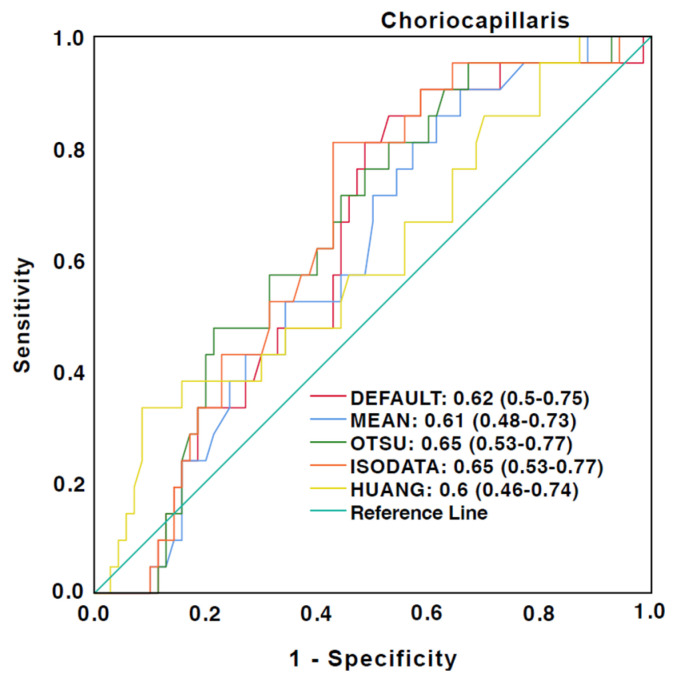
Receiver operating characteristics (ROC) curves for discrimination of diseased eyes from the healthy control group in the choriocapillaris slabs. The caption shows the area under the curve (AUC) values and the 95% confidence interval.

ROC curves for the discrimination between healthy eyes and eyes affected by DR, AMD, Uveitis, and RVO are illustrated in Figure 4 and Figure 5. A good ability for discrimination between healthy and diseased eyes was detected in the full retina slabs for all algorithms used. The highest AUC values were observed with Huang and Mean (Figure 4). However, a poor ability for discrimination was observed using the choriocapillaris slabs. The highest AUC values were detected with Otsu and ISODATA, while Huang had the lowest AUC values (Figure 5).

## 4. Discussion

In the present study, we compared five different automated thresholding algorithms to calculate the VD in OCTA images of the macula in full retina and choriocapillaris angiograms of eyes of patients with DR, RVO, Uveitis, AMD, and healthy eyes. We applied an LD-F2-analysis, intra-algorithm reliability, inter-algorithm agreement, and ability to discriminate between healthy and diseased eyes in commonly used auto-threshold methods: Default, Huang, ISODATA, Mean, and Otsu as implemented in ImageJ for image processing. As OCTA gains more and more importance in clinical routine, as well as in research, standardized as well as reliable techniques and processing methods are needed in order to restore comparability. Especially as VD is proposed as a new possible surrogate endpoint for clinical trials, it is essential to fully understand and compare clinical as well as technical aspects that may interfere with standardized measurements [21]. Even though VD in OCTA is known to have a good intra- and inter-operator repeatability when we use the same angiocube of the same device, recent studies have proven the dependence on different clinical factors, as well as differences in acquisition and the post-processing methods [11,22]. This includes significant differences in VD calculations based on the applied thresholding strategy [8,11,23,24]. Terheyden et al. found that automated algorithms outperform manual methods on 3 × 3 mm OCTA images to quantify macular perfusion. In addition, they emphasize the need for international standardization in clinical use [12]. A study by Arrigo et al. examined 13 automated algorithms for superficial as well as deep capillary plexus and choriocapillaris slabs. However, the cohort (30 eyes) was relatively small, and they only focused on healthy eyes. The best performing methods for binarization were Huang, Li, Mean, and Percentile, with overall good results [25]. Rabiolo et al. have stressed that studies adapting VD as an outcome should not rely on a normative database [11]. We aimed at evaluating VD more in depth by focusing on specific macular diseases. Diabetic retinopathy, AMD, uveitis, and RVO make up for more than 90% of macular diseases, in which a macular edema results in visual impairment and patients need recurrent intravitreal treatment. Microvascular changes are characteristic of all those four disease entities as microvascular abnormalities can be found in the retina as well as the choriocapillaris [26].

Our study found binarization results estimated with the five algorithms not to be interchangeable (*p* < 0.001), and that inter-algorithm agreement for image binarization was low. The results are consistent with existing data that have focused on other ophthalmological conditions [11,12,27].

Intra-algorithm reliability values range from excellent to poor and depend on the applied algorithm and examined retinal layer. For full retina slabs, reliability was excellent to good, except for eyes with AMD and not including Huang, which was poor to not reliable. Reliability results for the choriocapillaris slabs were moderate (Huang) to poor in healthy eyes and good to excellent in eyes with retinal disease. The poor results for healthy eyes are in line with a study by Laiginhas et al., which found significant advantages using local compared to global thresholding methods for binarization of the choriocapillaris angiograms [28]. Previous studies found local thresholding methods such as Phansalkar preferable to global automated methods for the segmentation of the choriocapillaris. Relying on the microvascular architecture of the choriocapillaris, local thresholding strategies lead to more promising results [22,29,30]. However, it remains unclear why the global thresholding algorithms used in the present study worked so much better with regard to reliability in diseased eyes.

The ability to discriminate between healthy and diseased eyes was good in all algorithms for full retina angiograms, and poor for the choriocapillaris slabs. Especially Mean and Huang showed good performances for the retina. Overall, the Mean algorithm detected sufficient values for discrimination, had good reliability and an ability for discrimination on full retina angiograms using the Copernicus Revo NX130 device. This corresponds to previously published data, supporting the theory that the Mean algorithm is a promising automated thresholding algorithm [12,25]. The Huang algorithm also had a good ability for discrimination of the full retina slabs but lacked reliability results. Default and ISODATA showed similar results in our study, which is based on the fact that the former is a slight modification of the latter.

In the future, volume rendered, 3D OCTA assessments will be interesting approaches for a more functional analysis. This method has been applied for a couple of conditions already and seems to be a reliable method for certain study designs [31,32]. However, as far as we know, choroidal sublayer 3D volume angiograms have not been studied yet. This might be an interesting approach for future studies.

Limitations of this study include its retrospective character and the relatively small number of eyes, which led to limited statistical testing such as for age-adjusted statistical comparison. In addition, there is no comparability and evaluation across different OCTA devices. Furthermore, we studied VD in full retina and choriocapillaris OCTA slabs. Other angiogram levels such as a superficial or deep capillary plexus might lead to different results. It is known that vessel density values depend on the device, angiocube size, image averaging, and post-processing methods. Therefore, our data only provide information in this specific setting. From a clinical perspective, we did not account for previous intravitreal medication in diseased eyes. As drugs such as inhibitors of the vascular endothelial growth factor (anti-VEGF) or steroids affect vascular density in the long run, our study cohort might be quite heterogenous. Moreover, the reaction of the vasculature in the different disease entities to those drugs varies [33].

In conclusion, when processing angiograms taken with the Copernicus Revo NX130, automated thresholding algorithms should be preferred for the binarization of full retina angiograms in eyes with DR, AMD, Uveitis, and RVO. When it comes to the choriocapillaris, other approaches should be considered. The Mean algorithm seems to be the most promising candidate for further prospective investigations.

## Figures and Tables

**Table 1:** Demographic and main clinical data of study population.

Parameters	Overall	Controls	Diseased	DR	AMD	Uveitis	RVO
No. Patients/Eyes	51/91	21/21	47/70	12/23	14/23	13/15	8/9
Laterality, left *n* (%*)*	48 (52.7%)	10 (47.6%)	38 (54.3%)	12 (52.2%)	12 (52.2%)	9 (60%)	5 (55.6%)
Age (years), mean ± SD	70.5 ± 11.7	67.1 ± 10.6	71.5 ± 12	69.8 ± 9.7	79.8 ± 5.9	63.1 ± 14.7	68.6 ± 12.4
Sex, female *n* (%)	27 (52.7%)	7 (33.3%)	41 (58.6%)	14 (60.9%)	14 (60.9%)	8 (53.3%)	5 (55.6%)
Lens status, phakic *n* (%)	35 (38.5%)	13 (61.9%)	22 (31.4%)	9 (39.1%)	7 (30.4%)	0 (0%)	6 (66.7%)
VA (logMAR), mean ± SD	0.30 ± 0.32	0.09 ± 0.12	0.37 ± 0.33	0.33 ± 0.22	0.34 ± 0.34	0.27 ± 0.22	0.69 ± 0.22
IOP (mmHg), mean ± SD	14.7 ± 3.5	13.5 ± 3.8	15 ± 3.5	16.5 ± 3.0	14.8 ± 3.2	14.5 ± 3.7	12.8 ± 4.2
AL (mm), mean ± SD	23.4 ± 1.0	23.5 ± 1.1	23.4 ± 0.9	23.4 ± 1.0	23.6 ± 0.8	23.2 ± 1.0	23.1 ± 0.5
CRT (μm), mean ± SD	272.1 ± 72.7	258.3 ± 33.3	276.2 ± 80.6	249.9 ± 40.2	260.1 ± 47.1	327.5 ± 127.3	299.0 ± 92.8

AL: axial length; AMD: age-related macular degeneration; CRT: central retinal thickness; DR: diabetic retinopathy; IOP: intraocular pressure; logMAR: logarithm of the minimum angle of resolution; RVO: retinal vein occlusion; SD: standard deviation; VA: visual acuity.

**Table 2:** Reliability analysis of the full retina slabs. The intraclass correlation coefficient and the respective 95% confidence interval are reported.

	Controls	Diseased	DR	AMD	Uveitis	RVO
Default	0.909 (0.801–0.963)	0.887 (0.829–0.927)	0.948 (0.895–0.977)	0.652 (0.253–0.855)	0.959 (0.903–0.985)	0.953 (0.854–0.989)
Huang	0.784 (0.525–0.913)	0.481 (0.216–0.667)	0.930 (0.858–0.969)	0.603 (0.146–0.870)	0.642 (0.149–0.870)	−1.069 (−5.466–0.492)
ISODATA	0.921 (0.826–0.968)	0.895 (0.841–0.932)	0.948 (0.895–0.977)	0.676 (0.305–0.865)	0.960 (0.906–0.986)	0.958 (0.869–0.990)
Mean	0.898 (0.777–0.959)	0.899 (0.848–0.935)	0.945 (0.889–0.976)	0.672 (0.296–0.864)	0.950 (0.881–0.982)	0.963 (0.883–0.991)
Otsu	0.924 (0.834–0.969)	0.894 (0.841–0.932)	0.950 (0.898–0.978)	0.683 (0.319–0.868	0.959 (0.902–0.985)	0.956 (0.862–0.989)

AMD: age-related macular degeneration; DR: diabetic retinopathy; RVO: retinal vein occlusion.

**Table 3:** Reliability analysis of the choriocapillaris slabs. The intraclass correlation coefficient and the respective 95% confidence interval are reported.

	Controls	Diseased	DR	AMD	Uveitis	RVO
Default	0.322 (−0.488–0.726)	0.958 (0.936–0.973)	0.953 (0.902–0.979)	0.953 (0.899–0.981)	0.923 (0.816–0.972)	0.973 (0.914–0.993)
Huang	0.605 (0.132–0.840)	0.935 (0.902–0.958)	0.871 (0.734–0.944)	0.885 (0.752–0.952)	0.967 (0.921–0.988)	0.933 (0.790–0.984)
ISODATA	0.244 (−0.660–0.694)	0.96 (0.94–0.975)	0.951 (0.898–0.979)	0.962 (0.918–0.984)	0.926 (0.825–0.973)	0.976 (0.925–0.994)
Mean	0.485 (−0.131–0.792)	0.930 (0.894–0.955)	0.905 (0.804–0.959)	0.917 (0.821–0.965)	0.938 (0.853–0.977)	0.951 (0.848–0.988)
Otsu	0.407 (−0.301–0.760)	0.96 (0.94–0.975	0.956 (0.908–0.981)	0.960 (0.914–0.983)	0.952 (0.886–0.983)	0.970 (0.905–0.993)

AMD: age-related macular degeneration; DR: diabetic retinopathy; RVO: retinal vein occlusion.

**Table 4:** Bland–Altman analysis for inter-algorithm agreement in the full retina slabs for the entire study cohort.

Algorithm Comparison		Agreement (Bland–Altman Analysis)		
Algorithm 1	Algorithm 2	MD	LoA	Range
Default	Mean	−10.13	−4.87/−15.39	10.52
Default	Otsu	1.69	2.5/0.87	1.63
Default	ISODATA	1.25	2.03/0.48	1.55
Default	Huang	−11.24	−5.03/−17.45	12.42
Mean	Otsu	11.81	17.3/6.27	11.03
Mean	ISODATA	11.38	17.03/5.74	11.29
Mean	Huang	−1.11	2.84/−5.06	7.9
Otsu	ISODATA	−0.43	0.04/−0.9	1.3
Otsu	Huang	−12.92	−6.37/−19.47	13.1
ISODATA	Huang	−12.49	−5.89/−19.1	13.21

MD: mean difference; LoA: limits of agreement.

**Table 5:** Bland–Altman analysis for inter-algorithm agreement in the choriocapillaris slabs for the entire study cohort.

Algorithm Comparison		Agreement (Bland–Altman Analysis)		
Algorithm 1	Algorithm 2	MD	LoA	Range
Default	Mean	0.9	7.29/−5.5	12.79
Default	Otsu	−2.36	−0.76/−3.96	3.2
Default	ISODATA	−0.91	−0.76/−3.96	3.2
Default	Huang	1.93	9.32/−5.41	14.73
Mean	Otsu	−3.26	3.29/−9.81	13.1
Mean	ISODATA	−1.81	5.25/−8.87	14.12
Mean	Huang	1.03	5.23/−3.16	8.39
Otsu	ISODATA	1.45	2.82/0.09	2.73
Otsu	Huang	4.29	11.53/−2.94	14.47
ISODATA	Huang	2.84	10.82/−5.14	15.96

MD: mean difference; LoA: limits of agreement.

## Data Availability

Not applicable.
